# Community member perspectives from transgender women and men who have sex with men on pre-exposure prophylaxis as an HIV prevention strategy: implications for implementation

**DOI:** 10.1186/1748-5908-7-116

**Published:** 2012-11-26

**Authors:** Gabriel R. Galindo, J. J. Garrett-Walker, Patrick Hazelton, Tim Lane, Wayne T. Steward, Stephen F. Morin, Emily A. Arnold

**Affiliations:** 1 Center for AIDS Prevention Studies, University of California at San Francisco, 50 Beale Street, Suite 1300, San Francisco, CA 94105 USA; 2 Department of Psychology, University of San Francisco, 2130 Fulton Street, San Francisco, CA 94117 USA; 3 HIV Epidemiology Section, Office of AIDS, San Francisco Department of Public Health, 25 Van Ness Avenue, San Francisco, CA 94102 USA

**Keywords:** Men who have sex with men (MSM), Male-to-female (MTF) transgender women, HIV/AIDS, Pre-exposure prophylaxis (PrEP), Qualitative research, Health disparities

## Abstract

**Background:**

An international randomized clinical trial (RCT) on pre-exposure prophylaxis (PrEP) as an human immunodeficiency virus (HIV)-prevention intervention found that taken on a daily basis, PrEP was safe and effective among men who have sex with men (MSM) and male-to-female transgender women. Within the context of the HIV epidemic in the United States (US), MSM and transgender women are the most appropriate groups to target for PrEP implementation at the population level; however, their perspectives on evidenced-based biomedical research and the results of this large trial remain virtually unknown. In this study, we examined the acceptability of individual daily use of PrEP and assessed potential barriers to community uptake.

**Methods:**

We conducted semi-structured interviews with an ethnoracially diverse sample of thirty HIV-negative and unknown status MSM (n = 24) and transgender women (n = 6) in three California metropolitan areas. Given the burden of disease among ethnoracial minorities in the US, we purposefully oversampled for these groups. Thematic coding and analysis of data was conducted utilizing an approach rooted in grounded theory.

**Results:**

While participants expressed general interest in PrEP availability, results demonstrate: a lack of community awareness and confusion about PrEP; reservations about PrEP utilization, even when informed of efficacious RCT results; and concerns regarding equity and the manner in which a PrEP intervention could be packaged and marketed in their communities.

**Conclusions:**

In order to effectively reduce HIV health disparities at the population level, PrEP implementation must take into account the uptake concerns of those groups who would actually access and use this biomedical intervention as a prevention strategy. Recommendations addressing these concerns are provided.

**Electronic supplementary material:**

The online version of this article (doi:10.1186/1748-5908-7-116) contains supplementary material, which is available to authorized users.

## Background

‘A pill a day reduces risk for HIV infection’[1]. Published in November 2010, this San Francisco Department of Public Health press release headline trumpeted a new era of human immunodeficiency virus (HIV) prevention as the results of a recent international randomized clinical trial (RCT) demonstrated that daily use of a combination antiretroviral pill (Truvada®) reduced HIV infections by 44%, overall, among HIV-negative status men who have sex with men (MSM) and male-to-female transgender women[2]. The trial, known as the iPrEx Study, assessed the efficacy of pre-exposure prophylaxis (PrEP) for HIV prevention when given as part of a comprehensive package of prevention services. The policy implications of the iPrEx Study data, promoting the use of PrEP to prevent HIV transmission at the population level, are being widely discussed[3–5]. However, before addressing the important issues of cost and delivery systems, even more fundamental implementation questions remain about community acceptability. Would those at highest risk be willing to take a pill a day? How does this approach fit with the way people think about their risk of HIV infection? In this study we explore, through semi-structured interviews with MSM and transgender women in three California counties, how some of these individuals understand the issues posed by PrEP and put forth relevant implementation recommendations.

Globally, there are 2.7 million new HIV infections annually; with the rate of new transmissions outpacing the rate at which HIV-positive diagnosed individuals enter treatment[6]. In this global epidemic MSM and transgender women who have sex with men bear a major portion of the burden[7, 8]. In the United States (US), there are currently over one million persons living with HIV/AIDS, with MSM populations constituting over half (53%) of new infections annually[9]. In California, MSM represent 61%, 72%, and 73% of living HIV/AIDS cases in Alameda[10], Los Angeles[11] and San Francisco[12] counties, respectively. Among transgender women, a recent review estimated HIV prevalence in the US between 11% and 28%[13]. Overall, the incidence of HIV in the US has remained stable in recent years; however, MSM and transgender women are the only groups for which HIV infection and AIDS diagnosis rates continue to rise[6].

The National HIV/AIDS Strategy for the US reports that ethnoracial health disparities in HIV infections exist as Black and Latino MSM are disproportionately affected by the epidemic, and increased attention to these populations is needed[9, 14]. A recent review of behavioral HIV interventions among MSM noted that while continued research is needed to identify which specific strategies are most effective in reducing transmission, and which intervention components are most effective in influencing those behaviors, US federally funded programs have had an effect in reducing HIV transmissions[15]. In fiscal year 2010, US Department of Health and Human Services domestic spending on direct prevention, care, and treatment of HIV/AIDS totaled nearly $9.7 billion, with 70% of those funds going toward direct client services through the Centers for Medicare and Medicaid[16]. Even in an era of fiscal conservatism, the US government has committed itself to supporting HIV prevention programming and services, particularly for those communities most impacted[17].

### The potential of PrEP as an HIV prevention tool

For sexually active individuals, the use of male condoms has remained the most cost-effective and readily accessible prevention tool for nearly three decades. While condom promotion interventions alone have had success in reducing HIV infections, new biomedical strategies for preventing transmission, including male circumcision, vaccines, topical microbicides, and the use of oral anti-retroviral (ARV) medication as prophylaxis, have increasingly been under study[18]. Recent work has acknowledged the role of comprehensive services in decreasing HIV disparities among MSM[19, 20]. According to the Centers for Disease Control and Prevention (CDC), PrEP is an HIV prevention method that requires HIV-negative individuals to take a daily pill to reduce their risk of becoming infected[21]. To date, three studies of PrEP reported successful reductions in HIV infections among heterosexual men and women[22]. The iPrEx Study, which included MSM and transgender women, remains the sole PrEP trial shown to be effective in populations who would benefit most from implementation in the context of the US HIV epidemic. A total of 2,499 individuals, 99% MSM and 1% transgender women, from six nations participated in the eleven-site iPrEx Study[2, 23]. The study design consisted of all participants receiving a comprehensive package of ongoing prevention services and health monitoring, including: intensive safer sex counseling, frequent dosing of condom promotion and distribution, screening of sexually transmitted infections (STIs) every 24 weeks or when warranted, monthly HIV testing, and select laboratory examinations.

Results from the study demonstrated an overall 44% (95% CI: 15, 63) reduction in HIV infection. Among those whose self-reported adherence was 90% or more, risk of HIV infection was reduced by 73% (95% CI: 41, 88). Initial data gauging attitudes of PrEP acceptability among MSM populations were conducted prior to the release of iPrEx Study results, and did not include transgender women. These findings have indicated a general lack of knowledge surrounding PrEP, but a willingness for utilization once aware of the potential for HIV prevention[24, 25]. One study, which presented 25 HIV serodiscordnant MSM couples with a hypothetical 90% PrEP efficacy rate, gave unique insights into factors that could influence uptake within a partner relationship, but was limited in extending these findings to those not in a partner relationship[26].

While literature on acceptability among ethnoracial minorities and transgender women community members is scarce, two studies since the release of the iPrEx results have shown that interest and use of PrEP among MSM varied across populations. One study, which sampled MSM engaged in online networking, noted that awareness of PrEP was limited one month after the iPrEx data were released and that utilization was extremely rare, despite the fact that MSM who reported high-risk behaviors were interested in using PrEP[27]. The other study, based in Australia, reported that high-risk MSM (*i.e.*, those reporting unprotected anal intercourse with another man) indicated a willingness to use PrEP; however, factors that would either increase or decrease their willingness were not ascertained in that quantitative investigation[28]. The goal of this study is to explore the factors surrounding PrEP acceptability among community members from populations disproportionately affected by the US HIV epidemic.

## Methods

Previous international qualitative work with MSM populations has been useful in providing nuanced understandings of sexuality, risk behaviors, social networks, and gay community life in relation to HIV surveillance and research[29–32]. We selected a qualitative approach so that salient concepts and themes relevant to the study goal could be developed[33]. That is, building from previous studies that quantified respondents theoretical use of PrEP in their sexual lives, we selected an inductive approach so that participants could broadly explore social and cultural influences that may play a role in their decision to use or not to use PrEP as an HIV prevention strategy. More so, this approach allows for participants to reflect on their intrapersonal experiences with sexual partners, the strategies they currently incorporate to reduce their risk behaviors, and to consider the potential impact that PrEP utilization may have on their fellow community members; information which scientists, providers, and policy makers may use to consider best strategies for implementation among target population members.

### Eligibility and recruitment

Thirty MSM and transgender women community members from Alameda, Los Angeles, and San Francisco counties (10 participants per county) were recruited to participate in semi-structured interviews. Potential participants learned of the study through word of mouth and flyer postings at social service organizations, community planning groups, and passive street recruitment. We purposefully oversampled ethnoracial minority MSM and transgender women to ensure that perspectives of these groups would be adequately represented in the dataset. Inclusion criteria were: self-identification as a man who has sex with other men or as a transgender woman; self-reported HIV-negative or unknown serostatus; self-report of at least one male sexual partner in the past year; and self-reported inconsistent condom use (*i.e.*, any report other than ‘always’ or ‘never’). We did not enroll participants who never used condoms, as we were interested in capturing perspectives from community members about risk compensation and whether men who were currently using condoms would continue to use them with PrEP.

### Data collection

Eligible participants provided verbal informed consent to participate in an interview that covered several domains (Table 1). A standardized statement regarding PrEP was read verbatim to each participant by the interviewer during data collection to ensure that all community members had the same basic level of information regarding the use of ARV’s as a prevention strategy (see Additional file1). This statement contained information on the iPrEx Study, including all prevention services offered in the intervention package as well as trial results. All interviews were digitally recorded and lasted up to one hour in length. Participants were compensated $40 for their time. The study protocol was approved by the UCSF Committee on Human Research.

**Table 1:** **Study interview guide topics**

Domain	Examples
**Demographics**	How would you describe your ethnicity?
	Are you currently employed? What is your job or profession?
	Do you currently have health insurance?
	How would you describe your sexual identity?
**Knowledge of PrEP and the iPrEx Study results**	Please tell me what you understand about PrEP. What have you heard, if anything?
	Were you aware of the results of the PrEP trial that were announced in November 2010? What did you think about the results when they were announced?
	Are people in your community talking about PrEP? What are they saying?
**Personal sexual-risk assessment**	How would you assess your risk for HIV infection?
	Why do you think you are [low/moderate/high] risk?
	How frequently do you test for HIV? What about other STDs?
**Willingness to take a daily ARV**	Would you be willing to take a pill on a daily basis? [if yes] Can you say why? [if no] Are there any circumstances under which you would be willing?
	How would taking a pill each day work in the context of your day-to-day life and routines?
**Ability to follow routine medical monitoring**	Would you be willing to test for HIV now, and then do regular HIV testing while you were taking PrEP?
	What would influence your ability to make and keep regular check-up appointments with your doctor?
**Acceptable side effects and potential long-term health risks**	Do you have any concerns about potential side-effects from taking PrEP? Can you give some examples?
	In your opinion, do the benefits of PrEP outweigh the potential risks? Can you say why?
**Associated cost and financial considerations**	Would you be willing to pay out of pocket expenses for PrEP? How much would you be willing to pay each month?
	If health insurance plans covered PrEP would you feel comfortable going to your doctor and asking for a prescription?
	How would you feel about having that information be part of your medical records and accessible to your insurance companies?
**Decision making about starting and stopping PrEP**	Under what circumstances would you might consider starting PrEP? What would be a motivator for you?
	Under what circumstances would you might consider stopping PrEP? What would make you not want to take it anymore?
**Perceived effects of PrEP in community**	Do you think the availability of PrEP will affect people’s willingness to use condoms?
	What would be a good way to make people in your community aware of PrEP?
	Overall, what is your opinion of PrEP as a method of HIV prevention [both for you and for your community]?

### Data analysis

Using an approach rooted in thematic analysis[34] we incorporated several qualitative data analysis techniques, including: inductive analysis, cross-case analysis, and analytical coding of textual data. Our thematic analysis process was informed by established behavioral theories used in HIV prevention, including: the health belief model (which notes that individual health behavior is governed by perception of personal susceptibility to disease, severity of disease, perceived efficacy of behavior in dealing with disease, and perceived barriers to adopting behaviors)[35]; the social ecology theory of behavior (which notes that health behavior is influenced by various physical and social conditions within environments on the person's well-being)[36]; and the theory of planned behavior (which notes that health behaviors are determined by an individual's health beliefs, attitudes, personality, social norms, and willingness to comply with those norms)[37]. Initial inductive analyses involved discovering emergent themes and patterns within the dataset to develop a project codebook[38]. From this preliminary codebook, code names and definitions evolved to match emerging data during iterative analyses of the interviews by project staff. Through reading and coding four common transcripts, coder agreement reached 90%, at which point the raters completed final coding of the dataset. Memos were also written to build theory about coding decisions and cross case analysis. Two field research team members met regularly to build coding consensus, to become familiar with participant narratives, to contextualize discrepancies, and to make coding and cross-case analysis decisions of newly uncovered themes. Quotes selected for inclusion reflect the experiences and views of participants, and were chosen based on their relevance to the study goal. Atlas.ti software was used to assist in data management throughout the analysis process[39].

## Results

Table 2 depicts key participant characteristics, by county. Participants ranged between 21 to 58 years of age. Overall, approximately three-quarters of our sample stated that they are employed, two-thirds reported some type of health insurance, and all but one had completed high school, suggesting a relatively educated sample. Although many expressed personal reservations, 23 of the 30 participants (76%) indicated that they were willing to consider the use of PrEP.

**Table 2:** **Community member characteristics, by county (n = 30)**

	Alameda (n = 10)	Los Angeles (n = 10)	San Francisco (n = 10)	Total sample (n = 30)
**Mean age (SD)**	32 (8.74)	32 (6.16)	44.2 (10.83)	36.07 (10.30)
**Employment Status**				
Employed	8	10	4	22 (73%)
Unemployed	2	0	6	8 (27%)
**Education**				
<High School	1	0	0	1 (3%)
HS/GED	2	2	2	6 (20%)
Some college	6	3	6	15 (50%)
College degree	1	5	2	8 (27%)
**Ethnoracial identity**				
Black	6	0	7	13 (43%)
Latino	2	8	0	10 (33%)
Mixed	1	0	0	1 (3%)
White	1	2	3	6 (20%)
**Gender**				
Male	8	7	9	24 (80%)
Female^a^	2	3	1	6 (20%)
**Health Insurance**				
None	3	3	1	7 (23%)
Public	2	0	8	10 (33%)
Private	4	4	1	9 (30%)
Unspecified	1	3	0	4 (13%)
**Sexual Orientation**				
Gay	3	7	6	16 (53%)
Bisexual	3	0	0	3 (10%)
Other sexual minority identity^b^	2	0	3	5 (17%)
Heterosexual^a^	2	3	1	6 (20%)
**PrEP willingness**				
Willing to use	3	4	3	10 (33%)
Willing to use, if certain information is provided	4	5	4	13 (43%)
Not willing to use	3	1	3	7 (23%)

^a^ Transgender participants. ^b^ This category is inclusive of all other self-identified sexual orientation descriptors (*e.g.*, ‘queer’).

Three major themes emerged from the dataset. First, participants displayed a considerable lack of awareness and knowledge surrounding PrEP. Second, participants expressed ambivalence towards successfully integrating PrEP within their existing personal HIV prevention efforts. Finally, participants noted concerns about the rollout of PrEP, in that they either worried that the branding of PrEP as a prevention tool may leave out critical components of the trial intervention, or that the scale-up of PrEP within the healthcare system may exclude sub-populations of impacted communities. We include a summary of these findings in Table 3. Below, we contextualize each finding with reference to participant data. We acknowledge that within these themes there exist topics where participant beliefs surrounding PrEP acceptability were not always mutually exclusively. Thus, we categorized findings into selected themes based on the context in which the participant described particular phenomena.

**Table 3:** **Summary of community member perspectives on the uptake and implementation of PrEP at the population level**

Finding	Examples	Considerations and/or Implications for policy and practice
**Community members are unaware and/or unknowledgeable about PrEP**	Of those who reported no previous understanding of PrEP:	Need for materials or procedures within PrEP programs that can overcome historical mistrust of the medical system.
	· Expressed mistrust of medical system	· Consumer information that is simplified and clearly written so that local community members accurately understand PrEP findings.
	· Do not believe PrEP actually exists and/or do not believe it will work	· Enhanced efforts to disseminate findings to local communities. This is likely to include use of venues and media accessed by those communities.
	· Did not know that any studies of an HIV prevention pill were underway	
	Of those who reported previous knowledge of PrEP:	
	· Often confused PrEP with PEP	
	· Had incorrect information regarding clinical trials	
	· Do not see its purpose	
**Community members expressed mixed interest in receiving PrEP**	Of those who expressed interest in using PrEP:	
	· Believe it would help connect individuals to the healthcare system	· It is critical for information campaigns about PrEP to focus on:
	· Expressed that anything to help reduce HIV transmission is a good thing	· (1) its efficacy at preventing HIV infection;
	· Noted that PrEP already fit into their medical routine	· (2) its safety;
	Of those who reported conditional willingness to use PrEP:	· (3) the logistical ease of the regimen and associated medical monitoring; and
	· Reported various concerns about safety and side effects	· (4) programming, funding sources or opportunities that would make PrEP affordable for low-income populations.
	· Believe cost is a major barrier (both for themselves and others)	
	· Need more statistics and data to make a final decision	
	Of those who expressed minimal to no interest in receiving PrEP:	
	· Do not believe PrEP is effective enough and/or believed condoms were a better alternative	
	· Feel that monitoring of side effects is burdensome	
	· Stated that a once-a-day regimen is not realistic	
**Community members noted concerns regarding the rollout of PrEP**	Of those who noted concerns about PrEP as a package:	Critical to implement PrEP as part of comprehensive programs that combine daily pill regimens with other strategies, such as testing and counseling and behavioral intervention approaches.
	· Believe much disinhibition and risk compensation will occur in their communities	· Need to develop protocols that define clearly the roles that clinical providers and community-based providers will play in the provision of combination prevention strategies that include PrEP and more traditional prevention approaches (e.g., testing, behavior change).
	· Fear that ARVs would be prioritized over education, condom use, testing and counseling	· Inclusion of diverse viewpoints in decision making around funding priorities for HIV prevention dollars.
	· Feel that messaging will not reach the communities it needs to	
	Of those who noted concerns about PrEP accessibility:	
	· Expressed multiple fears of equity and access	
	· Questioned the roles and responsibilities of providers, health insurance and pharmaceutical companies in reducing health disparities	

### Finding one: Community members are unaware and/or unknowledgeable about PrEP

By and large, participants from all counties had never heard of PrEP. Statements such as ‘I do not know what PrEP is. I have no knowledge of that information’ (31, White MSM, LA) were prominent. For those who had heard of PrEP prior to the interview, correct information was strongly overshadowed by mis-communicated facts and confusion with other treatments, particularly with post-exposure prophylaxis (PEP)[40], ‘…There’s PrEP and PEP, I get those two mixed up’ (42, Black MSM, SF); ‘It’s like the morning after pill so you won’t get pregnant, I think that’s the same type of pill’ (45, Black MSM, SF). Indeed, the notion of a ‘morning after pill’ in recounting PrEP information was expressed by the majority of participants who considered themselves knowledgeable about PrEP, and suggests confusion in regards to how ARVs function in an increasingly biomedically-focused HIV-prevention field.

Participants who reported limited PrEP knowledge tended to also report mistrust of medical systems and pharmaceutical companies, as well as discrimination within the medical industry. In particular, these issues were salient for Black participants:

*‘Because I’m suspicious of the pharmaceutical companies, and—there are a lot of rumors about pharmaceutical companies. Then friends of mine, who are—especially African-American men, who are suspicious of clinical trials… so there are always issues about the Tuskegee syphilis trials, and there’s always this suspicion, especially among a lot of Black people I know, that these trials are just hidden ways of genocide, and things like that—and they don’t feel comfortable. I’m talking from the transgender thing, the transgender girls said they’re not comfortable with the way they [providers] look at them or treat them… And that’s sad. That’s really sad, especially here in San Francisco’ (54, Black MSM, SF).*

However, these sentiments were not limited by ethnoracial minority identity:

*‘Because I just feel more capable of taking care of my sexual health because I feel like my doctors are not capable of taking care of my sexual health. They’re uneducated, they’re ignorant, they’re very heterocentric, almost homophobic. It just doesn’t jive… I mean it was the US government that was infecting people with syphilis, gonorrhea, and other STIs to see what they’re gonna do. So I will never trust the state to invest itself in my health and my awareness, as a poor person’ (25, White MSM, Alameda).*

A minority of participants noted that they believed PrEP would be ineffective, ‘this pill is a good idea but I don’t think it’s gonna work…’ (44, Black MSM, SF). Others expressed that they did not see the purpose of PrEP, ‘Why take the medication if you don’t need to? If you’re having so-called safe sex with a condom? With the use of a condom, then there’s no need to ingest any medication’ (36, Latino MSM, LA). Nonetheless, upon hearing of the efficacy of the iPrEx Study results several participants felt that PrEP would make a ‘good back-up plan,’ (*e.g.*, in cases where a condom broke), but that more information was needed.

### Finding two: Community members expressed mixed interest in personally receiving PrEP

Given the format of the semi-structured interview, and recognizing that the interview was the first time that many participants learned of PrEP (or gained correct PrEP information), thoughts and perspectives regarding their personal willingness to use PrEP often shifted during the interview process, and related to six key factors: the individual’s sexual-risk assessment; beliefs that PrEP would connect them to care and treatment; knowledge of side effects; perceived effectiveness of the intervention; self-awareness of medication adherence; and cost.

With respect to personal risk assessment, several community members reflected on their preferences to not use condoms, as well as instances where they have previously engaged in risky sexual behaviors. These individuals believed that an intervention such as PrEP might fit well with their current behaviors:

*‘Well to be honest, I don’t like using condoms myself. If I took PrEP, then I still wouldn’t use a condom, but I would feel better taking—I would be less worried or paranoid about getting HIV by using PrEP than I would if I weren’t using PrEP,’ (48, Black MSM, SF).*

*‘The reason I would take it is because I know that sometimes my behavior does get risky, especially when I use drugs… so yeah, I absolutely would take anything to prevent anything if my judgment goes down the toilet and I don’t use protection,’ (29, Black MSM, Alameda).*

One major motivator for the uptake of PrEP, as described by our participants, is its potential to link individuals to care. For example, participants described how community members who have traditionally not accessed health services could be linked to prevention programs if a PrEP intervention were in place:

*‘And when it’s prescribed by a doctor, it’s part of your entire medical treatment… and so I think that’s important, and that’s good—yeah, we’re gonna prescribe this to you but we’re gonna also require you to have counseling and explain to you this and talk about abstinence and condom and low-risk and needle exchange and the nine thousand things that you should be thinking about, and not just pop a pill, have fun,’ (54, Black MSM, SF).*

In addition to having the potential to link individuals to prevention services, participants noted the potential of PrEP to link community members to treatment services if they were to seroconvert while on PrEP, ‘But the good part will be, if they will get infected, they will be linked to care more easily,’ (25, Latina transwoman, LA).

Importantly, participants’ concerns and knowledge of PrEP side effects played a major role in swaying their personal pendulum of willingness to take PrEP: ‘My decision would be all because of side effects,’ (32, Mixed-race MSM, Alameda). Given the mild side effects in the iPrEx study, there was a spectrum of opinions regarding what tolerable side effects may be. In general, if the side effects were not severe (*e.g.*, headache, nausea, loss of sleep) or faded over time then participants were more likely to report willingness to use PrEP. Some side effects that participants reported as ‘deal-breakers’ were long-term damage to kidneys, sexual dysfunction (*e.g.*, failure to maintain an erection), and ‘serious bone damage.’ A few noted fears of complications with existing morbidities:

*‘Then that’s another thing that would scare me. ‘Cause I’m also—I’m a diabetic. I have Type 2 diabetes, and I also have high blood pressure… Usually a lot of people who suffer from diabetes are already having kidney problems… If I take this medicine will it make it worse?’ (49, Black transwoman, SF).*

Still, there were a select number of participants who explained that ‘there’s gonna be side effects to everything, no matter what you do,’ (39, White MSM, SF), so they would be willing to take PrEP, as an intervention package, regardless of the side effects.

Participants also weighed the efficacy of PrEP in their willingness decisions. Those who focused on adherence and a 70% PrEP efficacy tended to speak more favorably of ARVs as prevention than those who focused on the overall 44% efficacy:

*‘Well, I think—I’m in a high risk, because I don’t like using condoms when I have sex also. So if its’ been proven to actually reduce to 70% of the people who took the PrEP drug, then I’d be willing to try it also. I mean, as long as there are no side effects or anything like that, you know?’ (48, Black MSM, SF).*

*‘Well I’m just saying, if you take something every day, and you only have a 44% chance of preventing, getting what it’s suppose to prevent, then we’re not even talking 50/50, we’re talking a little less than 50%, or a little more than 50% getting HIV. So it’s kind of grim,’ (59, White MSM, SF).*

When poised with the question of whether taking a daily pill would interrupt their regular health service schedule, those who had established routines exclaimed:

*‘No. I already see a doctor once a month. I see my psychiatrist once a month, and I see my primary care doctor once every six months, so I don’t have a problem, actually, adhering to a certain regimen,’ (48, Black MSM, SF).*

In fact, those who reported daily use of other medications such as high-blood pressure, psychiatric, or diabetes medications, as well as multi-vitamins and other supplements, tended to speak favorably on their personal willingness to use PrEP.

Others commented on the added responsibility of taking a daily pill, ‘it’s a little hard for me to do something on a regular basis, all the time, every time, at the same time,’ (46, Latino MSM, Alameda); which would be compounded by the ‘burden’ of ongoing regular medical monitoring. In light of this consideration, some participants alluded to the thought of occasional (*i.e.*, intermittent) PrEP use, as well as other dosing alternatives that could make adherence more acceptable:

*‘If it was something I could take monthly, PrEP would be amazing. Like first of the month when I get paid, I take a pill, awesome! I can calendar it out. But on a daily basis man, that would be difficult!’ (25, White MSM, Alameda).*

Our participants described that the next major barrier to PrEP uptake, both in terms of personal and community level perceptions, was that of cost. In discussing their financial threshold for PrEP, reported values ranged between $5 and $500 per month; with the majority of participants describing an average of $20 to $25 per month as a ‘reasonable’ amount. Some descriptive thresholds included, ‘the price of a cup of coffee,’ or ‘about the same as daily vitamins,’ and for those with insurance, ‘about an average drug co-pay.’ These amounts and associated willingness to use PrEP, however, were based on theoretical situations if cost was at least partially covered. The realities of PrEP payment and willingness to use varied by individual; ‘…because I’m poor, so I imagine it would affect the chance of me using it. And my friends are poor,’ (35, Latino MSM, LA). In one extreme case, a non-insured participant commented that without any type of insurance or financial assistance, the prospect of acquiring disease and receiving no-cost services would be most opportune for his situation:

*‘I would be very honest, but if you are asking me to pay $12,000 for a preventive pill, whereas if I get the virus, I go to a free clinic and I’m going to get all the drugs for free, as stupid as it sounds, I’d probably prefer to have the virus and have my drugs for free…’ (37, Latino MSM, LA).*

Even still, for some, the use of male condoms made for a less burdensome and more cost-effective choice.

*‘Using a condom is much better… because just no tests. I don’t have to go to the doctor every month, I don’t have to be tested. I don’t’ have to keep paying to get the medicine—It’s a hassle,’ (22, Black MSM, Alameda).*

### Finding three: Community members noted concerns regarding the rollout of PrEP

The ways in which PrEP will be presented to the community, and its potential to impact sexual-risk behavior, was of utmost importance to participants. Most in the study believed that their personal sexual-risk behaviors would not increase if they were to begin a PrEP regimen, ‘and I don’t think it would encourage me to engage in more high-risk behavior,’ (54, Black MSM, SF). However, participants expressed fears of disinhibition occurring among their peers at the population level:

*‘Well, in the community, the African American community and gay community, there’s not a whole lot of condom use going on anyway. So if they hear about this drug—it’s like, oh it prevents you from getting HIV! People are gonna run with that. It’s gonna be like-people will be tryin’ to get these pills like they get street drugs…. so it will lessen the condom use, definitely…’ (30, Black MSM, Alameda).*

*‘Oh my god! I think it would be a crazy thing. It would be like—people would be popping all these pills and not taking care of themselves… Like not using condoms, because they feel that, ‘oh I’m not going to get it, because I’m taking these pill,’ or ‘oh, I could have unprotected sex and go pop a pill’… I just feel that would happen in my community. Just crazy, just—oh ya,’ (25, Latina transwoman, LA).*

It should also be noted that a select number of college-educated participants reported that population-level disinhibition may be unfounded speculation. These individuals attributed their sentiments to their perception that condomless sex was already occurring within MSM populations:

*‘It’s been my experience that people’s willingness to use condoms has already decreased significantly, and so I don’t think that this is going to add to that decrease that much. It seems like this is a good response to behaviors that are already happening – the decrease of condom usage,’ (28, White MSM, SF).*

Many participants noted that the delivery of PrEP, as suggested in previously provided quotes, should be more than promotion of daily ARV adherence. Participants stated fears that the medication portion of a PrEP intervention would be prioritized over condom use and education. Some participants explained that sometimes ‘life shows up’ and that for those who are not connected to stable social support and clinical care services, ‘it’s not gonna be a real successful thing to get them into counseling,’ (47, Black transwoman, Alameda), because they are already lacking adequate access.

In highlighting the significance of the educational component of a PrEP intervention package, a younger San Franciscan, who happened to be a health educator, framed the discussion in a historical context:

*‘We did a great deal of education when the HIV crisis hit and we learned that condoms prevent HIV, and I don’t think that we continue to do that kind of education that happened. And so I think it just needs a massive education campaign and a community buy-in. I think that’s a large part of it, too, and the community doesn’t have that buy-in right now…’ (28, White MSM, SF).*

Participants often discussed the notion of equity and access in relation to health disparities, as well as the question of who should financially support an effective PrEP rollout. One participant exclaimed that the responsibility of marketing PrEP to populations affected by the epidemic should fall on pharmaceutical companies who manufacture ARVs, ‘I would personally say that whoever created this medication, if they’re going to charge $12,000… then they should also have money set aside to also target the communities,’ (36, Latino MSM, LA). Feelings of, ‘I think this would actually cause people to feel discriminated against, because it’s the people who can afford and the people who can’t,’ (42, White MSM, LA), were also common among the narratives. While many framed the issue of access along socioeconomic lines, the only participant to possess a doctoral degree framed the issue of equity in regards to the belief that resources will be put towards HIV uninfected persons and not to those living with HIV. ‘It seems as like [though] more of the research has been going to preventing it [HIV], and not so much the people who actually have it,’ (42, White MSM, LA). Another participant concisely expressed the response of others in the study, ‘So obviously, if this is going to exist for the community, then hopefully something will be done in which everyone can afford it—not just the wealthy or the upper middle class,’ (36, Latino MSM, LA).

Individuals stated that ‘high-risk folks,’ including those with multiple partners, those who dislike the use of condoms, those who practice serosorting (*i.e.*, choosing sexual partners based on their perceived HIV status), those who use illicit substances, and those who engage in sex work, would be good candidates for tailored PrEP marketing and implementation:

*‘I think—in looking at the big issue for me is, how do we—the prevention part. How do we help the prevention part of it? When people are still gonna make stupid decisions, which is, let’s call it what it is. And that’s the high-risk folks. Then this is gonna be something good for them… so I think something like this could benefit a low socioeconomic community like ours, low education—and low-educated not just college, but just on the issue of protection,’ (31, Latino male, LA).*

*‘But the people that really need it are the people that are living in SROs [single room occupancy; government-subsidized housing], on GA [general assistance], Social Security SSI [supplemental social security income] or whatever they’re on… But if they gotta pay for it, you’re just gonna be sitting there with a bunch of pills,’ (44, Black MSM, SF).*

By underscoring the significance of PrEP utilization and its importance in reducing sexual and gender minority health disparities, the following reflections of an older Black transwoman, who reported often engaging in unprotected receptive anal intercourse, puts into context the potential of translating iPrEx Study results into population-level settings:

*‘Well, I didn’t know, like I told you when I came in, much about it [PrEP]. I’ve heard about it. But I think I have a more clear understanding, and I think it would be something very good for the LGBT community. We as a people are very sexually active. That’s our stigma. We probably always will be. We have younger LGBT members coming up, we have trans, lesbians, gays or whatever, and I think this drug would be just what is needed to help our community, to save our community, and I’m very much interested in learning more about it. I really am… We do get individuals that need some prevention in life and want some type of prevention, and this particular drug seems like it will do, if only for the 44 percent. It will do what needs to be done. The rest is up to me and to each individual that takes it, to make sure that we’re following through with the counseling, following through with the doctor visits, just to make sure, or following through with changing our diet, our eating habits. And in cases of, it has to be a condom, making sure that we use a condom, until we can be conclusive that this drug can be taken without the use of a condom and still prove to be successful at lowering the cases of HIV and AIDS in our community,’ (47, Black transwoman, Alameda).*

## Discussion

Previous work surrounding the translation of iPrEx Study results into community settings noted that the effectiveness of using PrEP as an HIV prevention strategy could be offset by: low or intermittent use of ARVs, disinhibition and risk compensation, and/or minimal uptake and limited access to available medications[41–43]. Our results, which revealed a deficiency in knowledge surrounding PrEP and other biomedical interventions is consistent with these previous studies. Our data further build upon the innovative qualitative work of prior international studies with the population[29–32], and add to the context of misunderstandings of PrEP information in communities by noting a lack of accurate information dissemination within these populations, as well as much confusion with PEP treatments. Additionally, our findings complement previous work by reporting that barriers such as cost, access, safety, equity, and education influence individual perceptions about the efficacy of PrEP implementation at the population level. However, our results also show that motivators for PrEP adoption, such as increased protection against HIV and connection to the healthcare system, could ease uncertainties associated with PrEP uptake. As our data collection tool included a synopsis of the iPrEx Study, the majority of our sample underscored the fact that PrEP should be delivered in the way that it was tested in the clinical trial: as an integrated prevention package that is not simply taking a daily ARV, but inclusive of education, counseling, STI screenings, condom promotion, and side-effect monitoring. Also consistent with previous PrEP work, fears were expressed that the condom promotion component of the intervention package would fall out over time and that disinhibition would occur[44]; however, many of our participants believed counseling and educational components of a comprehensive package of services would help compensate for these tendencies.

Additional studies are underway to answer questions brought on by the iPrEx results, and to address concerns raised by our study participants. The HIV Prevention Trials Network is conducting an international intermittent PrEP use feasibility study[45]. In San Francisco, CA and Miami, FL demonstration projects examining PrEP administration in sexual health city clinics will begin in 2012[46]. A 72-week open label extension of the iPrEx Study is currently enrolling participants to assess the ongoing efficacy, safety and adherence of PrEP[47]. Understanding the perspectives of community members, like those of the present study, is imperative as these individuals will actually be the persons accessing and utilizing PrEP in the near future.

### Recommendations

First, the way in which public health professionals frame PrEP will be a key factor in its acceptability by communities most impacted by the epidemic. Eradication of infectious diseases cannot occur through microbial medications alone; and the reduction of HIV incidence and associated health disparities will most likely involve a combination of biomedical and sociobehavioral interventions[48, 49]. A critical component of this combination prevention approach will be the delivery of accurate information to communities that translates findings in a way that is factual and does not lead populations to jump to the wrong conclusions. However, thus far, delivery of truly accurate PrEP information has yet to reach those groups disproportionately affected by the HIV epidemic. For example, our dataset notes that marketing PrEP as ‘A pill a day to keep HIV away’[50] is not a completely accurate description of PrEP, and undermines community members’ ability to comprehend and accept the complexity involved with a complete PrEP intervention. One way in which professionals can enhance their ability to increase PrEP knowledge and community member buy-in would be the use of community mobilization strategies. Community mobilization creates social change by building awareness of critical health issues and empowers community members to take charge of their healthcare needs through a collective, engaging and iterative process[51]; and has been effective in other population-level HIV prevention efforts[52].

Second, the way in which providers package PrEP as an intervention will be a key factor in its success as a prevention strategy for those most vulnerable to HIV infection. Previous work underscores the belief that the delivery of PrEP at the population level should be a multi-level intervention that cuts across individual and structural ecological levels in order to have optimal impact[41, 42, 48]. While there is currently no minimum standard of care for the provision of PrEP in community settings, there is consensus that medical monitoring, STI and HIV testing, behavioral risk reduction counseling, access to medications, condom promotion and distribution, public health campaigns, and coordination with local stakeholders are all crucial components that will either hinder PrEP implementation or add to its success. The questions of who, what, where, and how PrEP will be delivered now arise. As noted by our study population, mistrust with medical settings still exists within communities impacted by HIV, and even those with access to providers expressed concern of homophobia and non-culturally competent staff in the medical field. The promise of PrEP to incorporate biomedical and sociobehavioral approaches make the use of implementation science an ideal conceptual framework from which to consider delivery at the population level, and to answer the pragmatic and logistical questions involved with its administration[53].

Finally, the way in which costs associated with PrEP are absorbed or subsidized by private insurance companies, federal and local government programs, and/or pharmaceutical companies will be a key factor in its ability to reduce HIV-related health disparities by curbing the epidemic among those most-impacted. In the US, off-label costs of Truvada® are approximately $11,000 per year, which far exceeds the threshold of acceptable financial burden reported by our study sample. An examination of remunerative potential, conducted prior to the iPrEx Study results, noted that given a 50% efficacy rate, PrEP at the population level would not be sufficient to meet the standard of US cost-effectiveness[54]. Moreover, the study suggests that in order for PrEP to be attractive it would need to be targeted within populations at greater risk for HIV infection, including young and high-risk MSM. The manufacturers of Truvada®, have recently received approval from the Federal Drug Administration for the use of Truvada® as prevention[55]. While this has hopeful implications for those community members with access to healthcare, as noted by participants, this could create a situation of the ‘haves and have-nots,’ with an unequal distribution of medication and services. In an ever-increasing, resource-limited HIV prevention field, officials must decide if combination prevention approaches are the best use of funds when other, more cost-effective, strategies already exist[56–58]. This is a particularly important consideration as providers attempt to balance biomedical-focused interventions, like treatment as prevention and testing and linkage to care, with prevention programs that focus on the social dimensions of HIV prevention efforts[59, 60].

### Limitations

While data from this qualitative investigation present new and insightful understandings that help frame the discussion of PrEP implementation at the population level, it possesses several limitations. First, we were only able to gather the perspectives of individuals in three California regions. Given this, we are unable to generalize to other geographic locations as the experiences of those from rural areas, or other metropolitan areas, may vary considerably from individuals in our sample. However, participants represent three counties in California most impacted by the HIV epidemic. Second, being able to contrast community member perspectives with those of medical providers would have added another level of insight into the implementation of PrEP; indeed, in light of this, medical provider insight has been reported elsewhere[61]. Finally, the interview format may have introduced bias by providing participants with information on PrEP. Despite these limitations, the current study provides innovative insight into community member perspectives of PrEP and informs future directions and implications for its implementation.

## Conclusions

The advancement of PrEP for use as a new weapon in the arsenal to combat HIV transmission is promising. As the policy discussion regarding PrEP implementation continues to evolve, key stakeholders should make certain that: the communities most impacted by the HIV epidemic are knowledgeable about PrEP; that questions and concerns regarding medical mistrust and side effects are addressed; that PrEP is accessible financially; and that PrEP is a complete intervention package inclusive of education, condom promotion and linkage to care. Even with clinically proven individual-level efficacy, if not packaged, implemented, and sustained properly, PrEP could increase HIV health disparities at the population level that it initially had the potential to eliminate.

## Authors’ information

At the time of the study all authors were members of the University of California at San Francisco AIDS Policy Research Center (http://ari.ucsf.edu/programs/policy.aspx). The AIDS Policy Research Center (APRC) seeks to improve international, national, state, and local responses to challenges posed by the HIV/AIDS epidemic by conducting timely policy research and facilitating progressive interaction between UCSF research and policy decision-making. Data presented is part of a larger APRC investigation of pre-exposure prophylaxis. GRG is currently a Research Specialist with the San Francisco Department of Public Health and JJGW is an Assistant Professor at the University of San Francisco.

## Electronic supplementary material

Additional file 1: Standardized statement regarding PrEP. (PDF 57 KB)

## Abbreviations

AIDS: Acquired immunodeficiency syndrome
ARV: Anti-retroviral
HIV: Human immunodeficiency virus
LA: Los Angeles CA
LGBT: Lesbian gay, bisexual, transgender
MSM: Men who have sex with men
PEP: Post-exposure prophylaxis
PrEP: Pre-exposure prophylaxis
SF: San Francisco CA
US: United States of America.
