# Supplementary material for: Community member perspectives from transgender women and men who have sex with men on pre-exposure prophylaxis as an HIV prevention strategy: implications for implementation
Source: Implement Sci. 2012 Nov 26;7:116. doi: 10.1186/1748-5908-7-116 (PMC3527231; doi:10.1186/1748-5908-7-116)
Supplement: Supplementary file 1 — Additional file 1: Standardized statement regarding PrEP. (PDF 57 KB) [file 13012_2012_549_MOESM1_ESM.pdf]

ADDITIONAL FILE 1

Title: Standardized statement regarding PrEP

Description: Excerpt script regarding PrEP given to participants during the interview; part of the interview guide.

- *PrEP stands for "pre-exposure prophylaxis," an HIV prevention strategy that involves taking one pill a day of the antiretroviral drug Truvada, along with the regular use of condoms during sex.*
- *One recent clinical trial – the iPrEX study – followed nearly 2500 men who have sex with men and transgender women in six countries and found that the group who received PrEP was 44% less likely to become infected with HIV.*
- *iPrEX also found PrEP recipients experienced few side effects in taking the drug.*
- *Given the iPrEX results, it is likely that PrEP will soon become more available to gay/bi men and trans women, either through community clinics like City Clinic in San Francisco or through one's personal primary care doctor.*
- *If a person is interested in taking PrEP, he/she will be asked to take Truvada every day, as well as use condoms during sex. Also, he/she will need to have regular follow-up visits with their doctor at least once every three months, which will include getting tested for HIV and monitoring any potential drug side effects.*
